# Tannin Composition of Cabernet-Sauvignon and Merlot Grapes from the Bordeaux Area for Different Vintages (2006 to 2009) and Comparison to Tannin Profile of Five 2009 Vintage Mediterranean Grapes Varieties

**DOI:** 10.3390/molecules16021519

**Published:** 2011-02-11

**Authors:** Kleopatra Chira, Bénédicte Lorrain, Isabelle Ky, Pierre-Louis Teissedre

**Affiliations:** Faculté d'Oenologie, Institut des Sciences de la Vigne et du Vin, UMR 1219 Œnologie, INRA, Université Victor Ségalen Bordeaux 2, 210 chemin de Leysotte, 33882 Villenave d'Ornon Cedex, France

**Keywords:** Cabernet-Sauvignon, Merlot, mediterranean varieties, grapes, proanthocyanidins

## Abstract

The proanthocyandin composition of skins and seeds of Bordeaux Merlot (M) and Cabernet Sauvignon (CS) grapes was evaluated by HPLC-UV-fluorescence for four consecutive vintages (2006 to 2009). The results indicated a strong vintage effect on the tannin profile of each variety. However, and in spite of the vintage effect, some tannin characteristics such as mDP, %G and %P allow discrimination of both Bordeaux varieties. The same analyses were carried out for the 2009 vintage of five Mediterranean grape varieties (Syrah, Grenache, Mourvedre, Carignan and Counoise). The results demonstrated differences among these five varieties. Syrah appeared to exhibit the highest concentrations of flavanol monomers and dimmers, especially in skins. The comparison study between Bordeaux and Mediterranean grape varieties for the same vintage (2009) revealed that mDP and %G for seed extracts were parameters specific to each vineyard area.

## 1. Introduction

The Bordeaux grape growing region, in France, is synonymous with some of the most prestigious wines in the World. The Bordeaux vineyards represent 115,000 hectares, 75% of which are planted with red grape varieties that result in the production of high quality wines. Merlot (M) is the most widely planted variety, it covers alone more than 62% of the total surface; it is the predominant variety in the Libourne region (Saint Emilion and Pomerol). Cabernet-Sauvignon (CS) is the second Bordeaux grape variety (25%) and it is the emblematic cultivar of the Medoc and Graves areas. Cabernet Franc being the third red grape variety (12%) is rather restricted to the Entre-deux-mers and Libourne vineyards [1].

After Merlot, Grenache is the second most planted French red grape variety. It is found exclusively in south of France and in the Rhône Valley, an area associated with other cultivars characteristic of Mediterranean vineyards such as Syrah, Mourvedre, Carignan and Counoise. The French Mediterranean vineyards cover all Mediterranean coastal departments including, from West to East, the Roussillon, Languedoc, Côtes du Rhône, Provence and Corse areas. These vineyards were progressively dominated by varieties introduced from Spain and Italy.

Wine organoleptic properties are largely related to phenolic compounds extracted from the grape during the winemaking process. Among them, flavonoids, including anthocyanins and flavan-3-ols, are the most important for wine quality. Anthocyanins are pigmented compounds responsible for the red wine colour and they are essentially located in grape skins. Flavan-3-ols exist not only as monomers but also as oligomers and polymers, called condensed tannins or proanthocyanidins. Proanthocyanin structures vary in the nature of their constitutive sub-units, mean degree of polymerization (mDP) and linkage position. Proanthocyanidins are located in all the parts of a grape cluster but skins contain lower amounts of proanthocyanidins than seeds and their structural characteristic also differ. Grape seed proanthocyanidins comprise only procyanidins [subunits constituted of (+)-catechin (C) and (-)-epicatechin (EC)], whereas grape skin proanthocyanidins include both procyanidins and prodelphinidins [subunits constituted of (-)-epigallocatechin (EGC)] [2,3]. Skin proanthocyanidins have a higher mDP and a lower proportion of galloylated subunits than seed ones.

Condensed tannins are grape-derived compounds of great importance to red wine quality due to their astringent, bitter properties [4,5] and their role in the long-term color stability [6,7]. Astringency and bitterness are two major characteristics in grape and wine quality definition. Astringency is a tactile sensation, whereas bitterness is a taste. The molecular size of proanthocyanidins affects their relative bitterness and astringency level [4,5,8,9]. Overall, monomers are more bitter than astringent, whereas the reverse is true in the case of large molecular weight derivatives. 

Proanthocyanidin content and composition in grape berries depend on different factors such as climatic and geographical conditions, cultivation practices as well as stages of ripeness. Moreover, grape variety has also an important contribution on grape phenolic contents and composition; definition of polyphenols content and composition may be specific to grape variety. Carménère grape seeds and skins, for example, presented a higher mean degree of polymerization, a higher percentage of galloylation, compared to Cabernet Sauvignon seeds and skins [10]. Monastrell grapes (skins and seeds) demonstrated a higher mean degree of polymerization than Syrah [11]. Another study revealed that the monomeric and oligomeric content in Tempranillo seeds was the lowest one when compared to Graciano and Cabernet Sauvignon seeds [12]. However, Tempranillo skins showed higher content of monomeric, oligomeric, and polymeric flavanols than both Graciano and Cabernet Sauvignon skins [12]. In the literature, data concerning variety effect on Bordeaux wine grape phenolic composition are largely absent. 

Considering the importance of phenolic composition, along with the insufficient published data for Bordeaux grape phenolic composition, the framework of this study was to assemble a database for the tannin composition of Bordeaux grapes from prestigious vineyards from the 2006 to 2009 vintages, and placing them in relation to other famous French grapes varieties. From this perspective, the proanthocyanidin contents of grape skins and seeds extracts from both Cabernet Sauvignon and Merlot varieties were evaluated on four consecutive vintages from the Bordeaux area. In parallel, skins and seeds tannin compositions of five different Mediterranean grapes varieties (Syrah, Grenache, Mourvedre, Carignan, Counoise) were assessed for the 2009 vintage. Indeed, both vintage and variety influences were evaluated on grape proanthocyanidin contents and characteristics.

## 2. Results and Discussion

### 2.1. Proanthocyanidin composition of Bordeaux CS and M grapes (vintages 2006 to 2009)

#### 2.1.1. Grape variety effect on proanthocyanin composition of Bordeaux grapes

The flavan-3-ol monomers (C, EC, ECG) and oligomers (B1, B2, B3, B4 dimers and a Cat-Cat-Epi trimer T) were identified and quantified at harvest in both seeds and skins in 2006, 2007, 2008 and 2009. Proanthocyanidin characteristics such as mean degree of polymerization (mDP), percentage of galloylation (%G) and percentage of prodelphinidins (%P) were also investigated for the monomeric/oligomeric tannins fractions of seeds and skins of both grape varieties.

In order to have a better overview of the grape varieties’ impact (*i.e.*, Cabernet-Sauvignon and Merlot) the data sets corresponding to each vintage were combined and submitted to a one-way ANOVA analysis with factor variety (CS and M).

**Figure 1:**
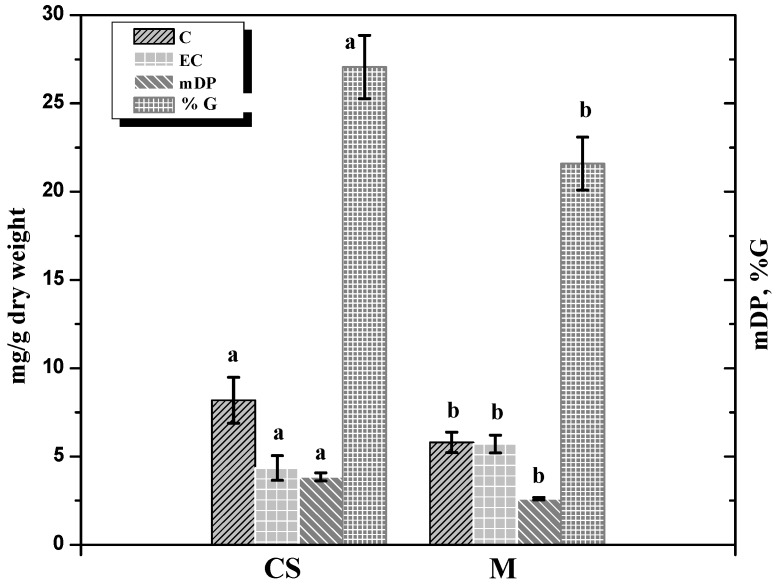
Variety influence on seed tannin composition.Values with different letters are significantly different (Tukey’s Test, p ≤ 0.05).

As seen in Figure 1, CS seed tannin extracts revealed higher catechin levels (8.18 mg/g), higher mDP (3.85) and % G (27.06) when compared to M seed tannin extracts (5.80 mg/g 2.61 and 21.58 respectively), in good agreement with Bozan *et al*.’s results [13]. In their study concerning seed polyphenol content of several grape varieties (in particular CS and M), they effectively indicate that differences were found in individual catechin contents among the two varieties. The main compound in seeds was catechin, with the exception of Merlot in which epicatechin was more abundant, as we also observed in our grapes in the 2009 and 2006 vintages (Table 1). Thus, the trend on individual proanthocyanidin concentrations among years may only be considered for catechin concentration as a constant indicator for distinguishing the two grape varieties. Independently of vintage, mDP and %G of seeds tannins appear to be appropriate tools in order to discriminate CS and M varieties. 

**Figure 2:**
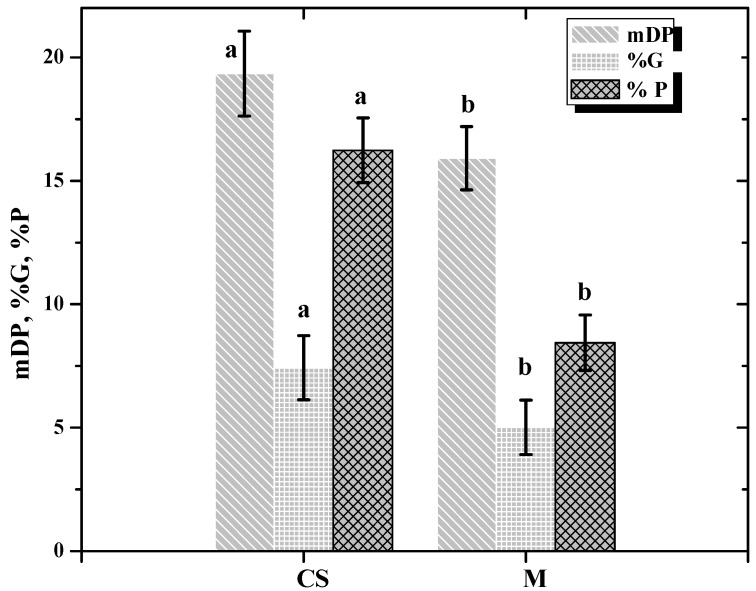
Variety influence on skin tannin composition. Values with different letters are significantly different (Tukey’s Test, p ≤ 0.05).

Regarding skin tannin extract composition, monomer and dimer concentrations were not affected by grape variety. On the other hand, mDPas well as % P and %G are influenced by variety (Figure 2). The CS skin tannin extracts are characterized by amDP (19.34), %G (7.42%) and %P (16.23%) values more important than those of M (*i.e.*, mDP = 15.9, %G = 5.01, %P = 16.23). Our results are consistent with previously reported mDP values of proanthocyanidins where grape seed tannin extract mDP values ranged from 2.7 to 18.6 [8,12,14], whereas skin polymeric proanthocyanidin mDP values ranged from 11 to 83, depending on the fractionating technique employed and on the grape variety and vintage [3,14].

Overall, the large majority of extractable flavan-3-ol monomers and dimmers are localized in the seeds for both varieties, as previously observed. In Syrah grapes [11], seeds show a higher proanthocyanidin level (2.28 mg/berry) than skins (0.39 mg/berry). Moreover, in another comparative study [15] focusing on a large panel of grape varieties (Cabernet Sauvignon, Carmenere, Marzemino, Merlot, Pinot Noir, Syrah and Teroldego), the highest amount of proanthocyanidins in all the grapes varieties was found in the seeds (93.8 to 681.3 mg/kg) and to a lesser extent in the skins (8.6-83.9 mg/kg).

**Table 1:** Seedsproanthocyanidinconcentrations and characteristics of Bordeauxgrape varieties. C, (+)-catechin; EC, (-)-epicatechin, ECG, epicatechingallate; B1, dimer B1; B2, dimer B2; B3, dimer B3; B4, dimer B4; T, trimer.

	Merlot-Seeds				Cabernet-Sauvignon-Seeds			
	2006	2007	2008	2009	2006	2007	2008	2009
C^*^	5.107b^**^	11.726c	3.618ab	1.806a	8.468a	17.415b	3.968a	2.048a
**StdErr**	0.842	0.707	0.254	0.206	0.984	3.612	0.208	0.346
**EC**	6.067b	10.652c	3.062a	2.354a	5.166ab	8.537b	2.065a	1.615a
**StdErr**	0.924	0.171	0.219	0.207	0.864	2.036	0.163	0.227
**ECG**	0.789a	2.622b	0.2689a	0.271a	0.470a	2.28b	0.205a	0.090a
**StdErr**	0.244	0.759	0.028	0.014	0.053	0.145	0.038	0.020
**B1**	3.179b	0.638a	0.143a	0.150a	4.386c	0.712b	0.112a	0.228ab
**StdErr**	0.307	0.031	0.019	0.058	0.295	0.181	0.018	0.092
**B2**	1.230a	3.778b	1.121a	0.588a	1.080a	6.727b	0.990a	0.791a
**StdErr**	0.143	0.391	0.127	0.039	0.149	1.740	0.118	0.264
**B3**	0.672b	1.283c	0.066a	0.323a	1.499b	1.675b	0.087a	0.275a
**StdErr**	0.112	0.105	0.008	0.064	0.119	0.475	0.024	0.080
**B4**	0.702a	2.748b	2.625b	0.384a	0.358a	3.742c	2.137b	0.527ab
**StdErr**	0.153	0.142	0.222	0.053	0.056	0.758	0.165	0.095
**T**	0.806a	3.477b	0.080a	0.120a	2.298b	0.091a	0.103a	0.044a
**StdErr**	0.116	1.114	0.038	0.035	0.488	0.031	0.020	0.009
**mDP**	2.407a	2.806b	2.962b	2.091a	3.620a	5.564b	3.377a	2.310a
**StdErr**	0.051	0.155	0.100	0.048	0.295	0.543	0.195	0.078
**%G**	9.279a	34.468c	19.633b	22.518b	18.700a	35.153b	25.174ab	27.160ab
**StdErr**	1.541	2.345	1.552	0.869	1.701	4.683	2.599	1.000

* concentrations in mg/g dw seeds; mDP, mean degree of polymerization; %G, percentage of galloylation. Std Err, standard error; ^**^ ANOVA to compare data, for each variety (M or CS) values with different letters within each row are significantly different (Tukey’s test, p < 0.05).

#### 2.1.2. Vintage effect on proanthocyanidin composition of Bordeaux grapes

In order to have a fine understanding of the influences of the vintage on the tannin compositions with a limited impact of the grape varieties, the data sets corresponding to each vintage were combined and submitted to a one-way ANOVA analysis (Table 1 and Table 2). An important vintage effect was observed for both varieties, not only on each studied tannin compound concentration but also on mDP for the two studied grape varieties, independently of grape part considered (seed or skin). Thus, in seeds, important differences between concentrations of individual compounds concentrations can be observed between vintages, leading in some cases to a 20-fold higher concentration (B1 concentrations in M between the 2006 and 2009 vintages). In particular, the 2007 vintage presented the most important concentrations of C, EC in all the samples (CS, M, seeds and skins) and the highest mDP in CS samples. Conversely the lowest phenolic compounds concentrations and mDP, values were recorded in 2009. This reflects the importance of the vintage on the tannin metabolism as already mentioned in our previous study [16].

**Figure 3:**
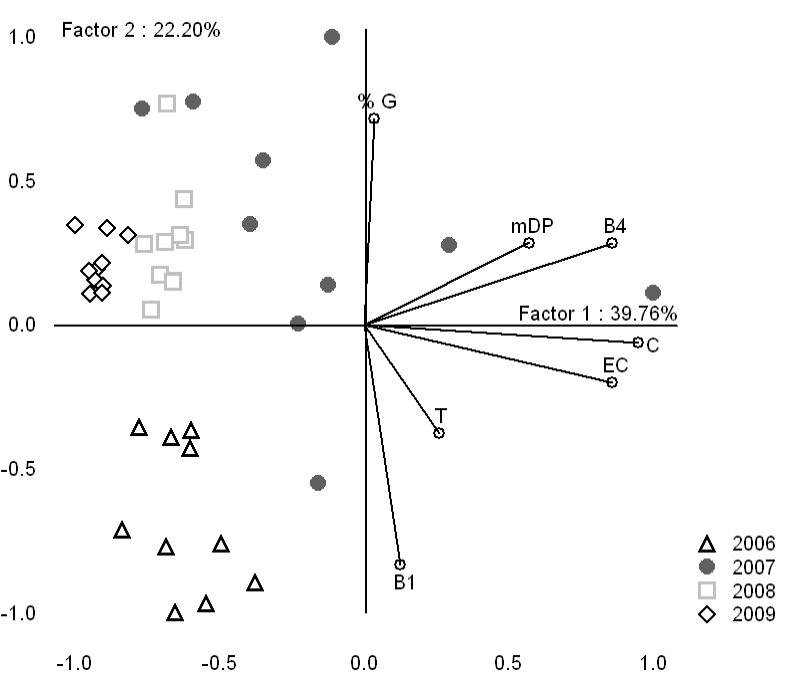
PCA representation of vintages (2006, 2007, 2008 and 2009) and the variables of seeds (C, EC, B1, B4, mDP and %G) in the plane defined by the two first principal components.

A PCA analysis carried out on the correlation matrix of the variables C, EC, B1, B4, mDP and %G allows the discrimination of vintages according to their seed tannin composition (Figure 3). The first Principal Component (PC1) is heavily negatively correlated with the levels of C, EC, B4, mDP, %G, whereas the second Principal Component (PC2) is strongly positively related to %G and negatively to B1. The 2007 seed tannin extracts show the greatest variability among the studied vintages (2006, 2008 and 2009). This is understandable considering the remarkable climatic conditions in Bordeaux during the 2007 vintage grape maturation season since the 2007 vintage was characterized by a strong water deficit and cool temperatures in August. These climatic conditions didn’t favor grape maturation and facilitated tannin accumulation. The seeds of both 2009 and 2008 are concentrated in the positive part of the second principal component and they are well separated, whereas 2006 seeds are found on the positive part of the PC2.

**Table 2:** Skinproanthocyanidinconcentrations and characteristics of Bordeaux grapes varieties. C, (+)-catechin; EC, (-)-epicatechin; B1, dimer B1; B3, dimer B3.

	Merlot-Skins				Cabernet-Sauvignon-Skins			
	2006	2007	2008	2009	2006	2007	2008	2009
C^*^	0.312b^**^	0.243b	0.025a	0.032a	0.127a	0.762b	0.045a	0.018a
**StdErr**	0.049	0.063	0.002	0.005	0.017	0.108	0.011	0.003
**EC**	0.031a	0.481b	0.014a	0.029a	0.098a	0.625b	0.036a	0.006a
**StdErr**	0.006	0.079	0.002	0.008	0.011	0.196	0.009	0.002
**B1**	0.015a	0.082a	0.074a	0.027a	0.034a	0.036a	0.070a	0.007a
**StdErr**	0.002	0.038	0.005	0.003	0.010	0.023	0.015	0.002
**B3**	0.014a	0.484b	0.070a	0.009a	0.085a	1.502a	0.158a	0.004a
**StdErr**	0.004	0.166	0.008	0.001	0.017	0.708	0.022	0.001
**mDP**	24.117c	15.113b	16.566b	3.910a	21.955b	26.623b	19.882b	3.97a
**StdErr**	1.997	1.970	1.823	0.331	1.597	3.762	2.299	0.316
**%G**	1.405a	1.398a	1.086a	21.360b	2.721a	4.388a	2.656a	23.800b
**StdErr**	0.225	0.183	0.118	1.427	0.287	0.698	0.330	2.660
**%P**	2.419a	3.528a	9.950b	22.730c	10.191a	11.012a	23.508b	25.880b
**StdErr**	0.266	0.643	0.716	2.284	1.217	1.813	1.628	2.400

* concentrations in mg/g dw skins; mDP, mean degree of polymerization; %G, percentage of galloylation; %P, percentage of prodelphinidins. Std Err, standard error; ^**^ ANOVA to compare data, for each variety (M or CS) values with different letters within each row are significantly different (Tukey’s test, p < 0.05).

### 2.2. Proanthocyanidin composition of Mediterranean grapes (2009 Vintage)

The second objective of the present paper was to place Bordeaux CS and M grapes varieties in relation to other well-known Côtes du Rhône red grapes varieties, especially Grenache, Syrah, Mourvedre, Carignan and Counoise. Table 3 and Table 4, respectively, indicate the 2009 seed and skin concentrations for each proanthocyanidin, as well as their characteristics. 

#### 2.2.1. Grape variety effect on the proanthocyanidin composition of Mediterranean grapes

In seeds, the analysis of variance revealed significant differences among the studied varieties on the levels of epicatechin, mDP and %G **(Table 3**). Mourvedre shows the lowest proanthocyanidin concentrations of all the tested Mediterranean varieties. On the other hand, Counoise grape seeds presented the highest content for all proanthocyanidins, but the difference was particularly significant in the case of epicatechin. In another study comparing Tunisian Syrah to Tunisian Carignan variety for the 2007 vintage [17], authors noted that flavonoid content varied with cultivar. They observed higher EC and B1 dimer concentrations in Syrah seeds than in Carignan ones, whereas the opposite was observed for C concentrations. In our study, the difference between varieties were only significant in the case of EC concentration, which was higher in the case of Syrah variety. These differences between studies underline the importance of the different terroirs and cultivation practices, but also of vintage on the tannin metabolism pathway. Regarding tannins characteristic, Mourvedre exhibited significantly higher mDP (4.6) and %G (51.9) than the other Mediterranean varieties.

**Table 3:** Seedproanthocyanidinconcentrations and characteristics of Bordeauxand Mediterranean grapes varieties for the 2009 vintage.

	CS^*^	M	Gre1	Gre2	Syrah	Carignan	Mour	Counoise
**C** ^**^	2.048a^***^	1.806a	2.330a	1.141a	1.807a	0.700a	0.433a	2.205a
**StdErr**	0.346	0.206	0.001	0.127	0.000	0.009	0.163	0.036
**EC**	1.615abc	2.354c	1.008abc	0.912abc	2.313abc	0.615ab	0.377a	2.516bc
**StdErr**	0.227	0.207	0.000	0.105	0.000	0.002	0.139	0.055
**B1**	0.228a	0.150a	0.145a	0.131a	0.129a	0.102a	0.081a	0.139a
**StdErr**	0.092	0.058	0.003	0.015	0.000	0.007	0.029	0.004
**B2**	0.791a	0.588a	0.432a	0.428a	0.338a	0.373a	0.183a	0.567a
**StdErr**	0.264	0.039	0.000	0.053	0.000	0.006	0.066	0.008
**B3**	0.275a	0.323a	0.259a	0.175a	0.136a	0.128a	0.112a	0.144a
**StdErr**	0.080	0.064	0.001	0.017	0.000	0.010	0.041	0.000
**B4**	0.527a	0.384a	0.189a	0.174a	0.153a	0.153a	0.110a	0.190a
**StdErr**	0.095	0.053	0.001	0.016	0.000	0.002	0.042	0.004
**mDP**	2.310ab	2.091a	2.732bc	2.600bc	2.242ab	3.106c	4.558d	2.112ab
**StdErr**	0.078	0.048	0.017	0.096	0.032	0.052	0.206	0.000
**%G**	27.160b	22.518a	37.485c	34.898c	35.269c	36.051c	51.884d	28.697abc
**StdErr**	1.000	0.869	0.856	1.448	1.499	0.085	0.708	0.361

^* ^ CS, Cabernet-Sauvignon; M, Merlot; Gre, Grenache, Mour, Mourvedre; C, (+)-catechin; EC, (-)-epicatechin, ECG, epicatechingallate; B1, dimer B1; B2, dimer B2; B3, dimer B3; B4, dimer B4; T, trimer; ^**^ concentrations in mg/g dw seed; mDP, mean degree of polymerization; %G, percentage of galloylation. Std Err, standard error; ^***^ ANOVA to compare data, for each variety (M or CS) values with different letters within each row are significantly different (Tukey’s test, p < 0.05).

As far as skins are concerned, the differences among the studied varieties are found in the concentrations of tannin monomers (C, EC), dimers (B1, B3) as well as the mDP and the %G (Table 4). An attentive look at this table reveals that Syrah exhibits significantly higher concentrations for all the investigated proanthocyanidins, while Grenache1 and Mourvedre exhibited both the highest mDP and %P. Concerning %G, Counoise skins display the highest value.

#### 2.2.2. Comparison of proanthocyanidin composition between Bordeaux and Mediterranean grapes

CS and M varieties present similar catechin concentrations in seeds as the Mediterranean varieties (Table 3). In the case of epicatechin, M exhibited high concentrations, equivalent to those of Syrah and Counoise. In other comparative studies [16,19,20] of Syrah and Grenache varieties with CS and M, no differences were found in monomer and dimer concentrations between varieties in seeds, a result which is in good agreement with our data. Regarding characteristic proanthocyanidins, our results are in contradiction with those of Cosme *et al*. [18]; we observe no significant difference in mDP values between CS - M (2.31 and 2.09 respectively) and Syrah (2.24). However, Grenache, Carignan and Mourvedre exhibit significant higher mDP than CS and M. For instance Mourvedre variety presented a mDP (4.58) and a % G (51.84) two times higher than CS variety (2.31, mDP; %G, 27.16) and M (2.09, mDP; %G, 22.52). In the case of seed %G, a cleaner pattern was observable since all the Mediterranean varieties exhibited higher values than CS and M varieties. Moreover, these differences were statistically different for all the samples, except for the Counoise variety. 

**Figure 4:**
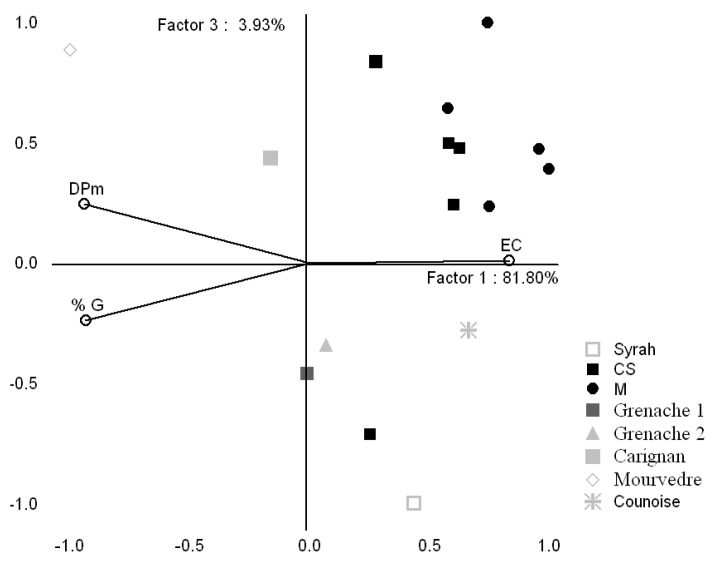
PCA representation of studied grape varieties for 2009 vintage and the variables of seeds (EC, mDP and %G) in the plane defined by the first and the third principal components.

In order to illustrate the significant seed differences among the above varieties a PCA was realized (Figure 4). The first Principal Component (PC1) is heavily negatively correlated with the %G and mDP, whereas EC is represented positively by the first and the third Principal; on the whole, M and CS samples are rather found on the right hand of the PC1 whereas the majority of Mediterranean grapes seeds samples are localized on the negative side of the PC3.

In skins (Table 4), Syrah shows almost 100 times and 30 times more B1 concentration than CS and M, respectively. In spite of this observation, CS and M present the same mDP than Grenache 2 variety (almost 4) and almost the same %G as Syrah variety. Thus no obvious trend allowing discrimination between Bordeaux and Mediterranean varieties was observed for either monomer and oligomer concentrations or for characteristic tannins, in good accordance with other studies [15,18].

**Table 4:** Skinproanthocyanidinconcentrations and characteristics of Bordeauxand Mediterranean grapes varieties for the 2009 vintage.

	CS^*^	M	Gre1	Gre2	Syrah	Carignan	Mour	Counoise
C^**^	0.018a	0.032a	0.144c	0.014a	0.276d	0.076b	0.035ab	0.030a
**StdErr**	0.003	0.005	0.007	0.001	0.016	0.014	0.008	0.002
**EC**	0.006a	0.029ab	0.056ab	0.001ab	0.079b	0.021ab	0.013ab	0.016ab
**StdErr**	0.002	0.008	0.004	0.000	0.059	0.005	0.002	0.001
**B1**	0.007a	0.027a	0.050a	0.017a	0.919b	0.037a	0.029a	0.021a
**StdErr**	0.002	0.003	0.008	0.001	0.062	0.007	0.010	0.001
**B3**	0.004a	0.009bc	0.021d	0.003a	0.027d	0.012c	0.005ab	0.003a
**StdErr**	0.001	0.001	0.003	0.000	0.002	0.003	0.001	0.000
**mDP**	3.970ab	3.910ab	5.682b	4.052ab	1.674a	1.366a	5.343b	2.816ab
**StdErr**	0.316	0.331	0.278	1.909	0.023	0.042	0.537	0.019
**%G**	23.800a	21.360a	29.863ab	28.299a	29.917ab	18.851a	18.317a	50.666b
**StdErr**	2.660	1.427	8.463	1.025	0.206	2.735	1.131	0.733
**%P**	25.880c	22.730bc	47.411d	16.15abc	11.269abc	8.550ab	52.120d	5.754a
**StdErr**	2.400	2.284	5.077	1.056	1.098	0.383	0.825	0.237

^*^ CS, Cabernet-Sauvignon; M, Merlot; Gre, Grenache; Mour, Mourvedre; C, (+)-catechin; EC, (-)-epicatechin; B1, dimer B1; B3, dimer B3; ^**^ concentrations in mg/g dw skins; mDP, mean degree of polymerization; %G, percentage of galloylation; %P, percentage of prodelphinidins; Std Err, standard error; ^***^ ANOVA to compare data values with different letters within each row are significantly different (Tukey’s test, p < 0.05).

**Figure 5:**
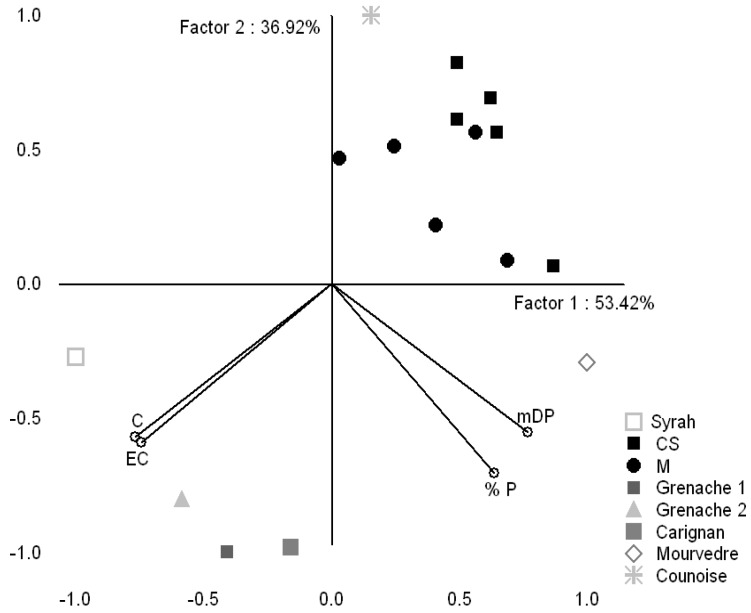
PCA representation of studied grapes varieties for 2009 vintage and the variables for skins (C, EC, mDP and %G) in the plane defined by the two first principal components.

The discrimination of the skin samples based on variety was achieved using the variables C, EC, mDP and %G (Figure 5). All the variables have a strong effect on the PC1 (mDP, %G in a positive way and C, EC in a negative way), for PC2 the variable with higher incidence (in a negative way) is the %G, followed by EC, C and mDP. The distribution of skin extracts showed that the majority of CS and M samples are on the right side of the first and on the positive side of the second PC. They are well separated from Mediterranean grapes which are located, principally on the negative side of the second component. 

## 3. Experimental

### 3.1. Reagents

Deionised water was purified with a Milli-Q water system (Millipore, Bedford, MA, USA). Acetonitrile (HPLC grade), ethyl alcohol (HPLC grade), methyl alcohol (HPLC grade), acetic acid, orthophosphoric acid, L-ascorbic acid, L-tartaric acid, hydrochloric acid, ammonia and sodium acetate were purchased from Prolabo-VWR (Fontenays/Bois, France). (+)-Catechin (C), (-)-epicatechin (EC), (-)-epigallocatechin (EGC), (-)-epicatechin-3-*O*-gallate (ECG), B1 [(-)-epicatechin-(4β-8)-(+)-catechin] and B2 [(-)-epicatechin-(4β-8)-(-)-epicatechin] were purchased from Sigma-Aldrich (Saint Quentin Fallavier, France). B3 [(+)-catechin-(4α-8)-(+)-catechin]; B4 [(+)-catechin-(4α-8)-(-)-epicatechin] and trimer (T) [(+)-catechin-(4β-8)-(+)-catechin-(4β-8)-(-)-epicatechin] were synthesised by the Laboratory of Organic and Organometallic Chemistry, Université Bordeaux 1, France [20].

### 3.2. Selection of experimental area and samples

The study was carried out on grape samples from five parcels located in the Bordeaux vine growing region in the south west of France for four consecutive vintages: 2006-2007-2008 and 2009. The parcels are situated in the Pauillac (P1), Margaux (P2), Saint Emilion (P3), Saint Emilion (P4) and Côtes de Bourg (P5) areas. The vineyards are all planted with *V. vinifera* L. cv. Cabernet Sauvignon (CS) and Merlot (M). In parallel, *V. vinifera* L. cv. Mourvedre, Counoise, Carignan, Syrah and Grenache berries (two samples) grown during the 2009 harvest season (Côtes du Rhône vineyard) were also used in this study. 

### 3.3. Samples collection and preparation

After harvest, seeds and skins were gently separated by hand, washed with distilled water, lyophilised for two days and then stored at −20 °C. Frozen seeds and skins samples were separately grounded in a ball grinder and the obtained powders were stored at −20 °C.

### 3.4. Proanthocyanidins analysis

#### 3.4.1. Fractionation of seeds and skinsproanthocyanidins

An aliquot (5 g) the obtained powder was extracted using acetone-water (70:30, v/v, 45 mL) followed by methanol-water (60:40, v/v, 45 mL). Both centrifugal supernatants were combined and evaporated to dryness under reduced pressure at 30 °C and then the residue was dissolved in water and freeze-dried to obtain 3.5 g of crude tannin extract as a powder. Each crude grape sample extract was prepared in duplicate.

*Seeds.* The crude seed tannin extract was first dissolved in distilled water (250 mL) containing 5% ethanol to help solubilisation at a concentration of 80 g/L. This solution was extracted three times with chloroform (250 mL) to remove lipophilic material and then the aqueous phase was finally extracted three times with ethyl acetate (250 mL) to obtain low molecular weight procyanidins (oligomeric tannins) in the organic phase. The ethyl acetate fraction was evaporated to dryness under reduced pressure at 30 °C; the residue was dissolved in waterand freeze-dried to yield 300 mg of monomeric-oligomeric proanthocyanidin seed extract as a dry powder used for HPLC-UV analysis.

*Skins*. Crude skin tannin extract (2.4 g) was dissolved in deionised water (10 mL) and then fractionated on Toyopearl TSK HW-50 (F) gel from Tosoh Corp. After loading the sample, the column (70 × 21 mm) was first washed with distilled water (200 mL) and then the monomers and tannin oligomers were eluted with methanol (900 mL). After evaporation of the organic solvent under reduced pressure at 30 °C, the residue was dissolved in water and freeze-dried to obtain 20 mg of skin tannin extract used for HPLC-UV analysis.

#### 3.4.2. HPLC Analysis of monomeric and oligomeric flavan-3-ols

The equipment used for the HPLC analysis consisted of a Finnigan UV-Vis detector (UV-Vis 200), a Finnigan autosampler and a Finnigan quaternary pump coupled to an Xcalibur data treatment system. The separation was performed on a reversed-phase Agilent Nucleosil C_18_ column (250 mm × 4 mm, 5 μm) at room temperature, with a flow rate set at 0.5 mL/min. The mobile phases were 50 mM dihydrogen ammonium phosphate adjusted to pH 2.6 with orthophosphoric acid (solvent A), 20 % solvent A with 80 % acetonitrile (solvent B) and 0.2 M orthophosphoric acid adjusted with ammonia to pH 1.5 (solvent C) at a flow rate of 0.5 mL/min. The initial conditions were set at 97 % of A and 3 % of B follow by a ternary mobile phase gradient : 97% A and 3% B at 5 min, 92% A and 8% B at 15 min, 0% A and 8% B at 18 min, 0% A and 13% B at 30 min, 0% A and 20% B at 55 min, 0% A and 25% B at 60 min, 0% A and 30% B at 70 min, 0% A and 80% B at 75 min, 0% A and 97% B at 80 min, 97% A and 3% B from 82 min to 84 min. Eluted peaks were monitored at 280 nm. Calibration curves were established at 280 nm using external standards, either commercial (C, EC, ECG, B1, B2,) or synthesized (B3, B4, T). Each sample was injected three times, the unknown concentrations were determined from the regression equations and the results were converted into mg by grams of dried seeds or skins. 

#### 3.4.3.HPLC-MSanalysis of mean degree of polymerization (mDP), %P, %G

The proanthocyanidin mDP, %G and %P were determined by a phloroglucinolysis reaction using the previously describe procedure [21]. LC-MS analyses of the reaction mixtures were performed on a Micromass Platform II simple quadruple mass spectrometer (Micromass-Beckman, Roissy Charles de-Gaulle, France) equipped with an electrospray ion source. The mass spectrometer was operated in negative-ion mode with the source temperature set at 120 °C, the capillary voltage set at 3.5 kV and the cone voltage set at −30 eV. HPLC separations were performed on a Hewlett-Packard 1100 series instrument (Agilent, Massy, France) including a pump module and a UV detector. Both systems were controlled by Masslynx 3.4 software. The elution profiles were recorded at 280 nm and the mass spectra were recorded between 50 to 1,500 amu. The separation was performed on a reversed-phase Waters XTerra RR C_18_ (100 mm × 4.6 mm, 3.5 μm) column at room temperature. The separation method uses a binary gradient with mobile phases containing 1% (v/v) aqueous acetic acid (solvent A) and MeOH (solvent B). The solvent gradient described below for oligomeric proanthocyanidins was applied at a flow rate of 1 mL/min. The elution conditions were: 5%B for 1 min, a linear gradient from 5 to 16%B in 1 min, a linear gradient from 16 to 22%B in 6 min a linear gradient from 22 to 35%B in 1 min, a linear gradient from 35 to 42%B in 7 min, a linear gradient from 42 to 100% B in 1 min. The column was then washed with 100%B for 3 minutes and re-equilibrated with 5%B for 4 min before the next injection. Proanthocyanidin cleavage products were estimated using their response factors according to a previous study [22].

### 3.5. Data analysis

Statistical data analysis was performed using Analysis of Variance (ANOVA) using Statistica V.7 (Statsoft Inc., Tulsa, OK, USA). Tukey’s HSD was used as comparison tests when samples were significantly different after ANOVA (p < 0.05) for chemical analysis. Principal Component Analysis (PCA) was used to examine any possible grouping of samples according to grape variety. PCA was performed on the correlation matrix using the attributes that differed significantly by ANOVA. In order to compare the grape variety and vintage effect on phenolic composition and tannin perception, the data sets corresponding to each vintage were combined and submitted to a two way ANOVA analysis with factor 1 grape variety and factor 2 vintage (2006, 2007, 2008 and 2009).

## 4. Conclusions

In this work, we have first investigated the influence of grape variety on proanthocyanidin composition of grape skin and seeds tannin extracts from Bordeaux grapes. We have demonstrated that in spite of a strong vintage effect, some proanthocyanidin specific criteria such as higher values of mDP and %G differentiate CS from M. The parallel analysis of Mediterranean grapes varieties in 2009 underlines that seed mDP and mostly %G were two distinctive tannin characteristics between Bordeaux and Mediterranean varieties. Principal component analysis (PCA) models were built to identify phenolic parameters exploitable to classify varieties and vintages.
